# Ultrasound-Assisted Extraction of Mangiferin from Mango (*Mangifera indica* L.) Leaves Using Response Surface Methodology

**DOI:** 10.3390/molecules19021411

**Published:** 2014-01-27

**Authors:** Tang-Bin Zou, En-Qin Xia, Tai-Ping He, Ming-Yuan Huang, Qing Jia, Hua-Wen Li

**Affiliations:** 1 Department of Nutrition and Food Hygiene, School of Public Health, Guangdong Medical College, Dongguan 523808, China; E-Mails: zoutb@163.com (T.-B.Z.); 2284@gdmc.edu.cn (E.-Q.X.); 1450@gdmc.edu.cn (T.-P.H.); 1314@gdmc.edu.cn (Q.J.); 2 Department of Sanitary Inspection, School of Public Health, Guangdong Medical College, Dongguan 523808, China; E-Mail: 2184@gdmc.edu.cn

**Keywords:** ultrasonic-assisted extraction, mangiferin, mango leaves, response surface methodology

## Abstract

Mangiferin is a xanthone widely distributed in higher plants showing antioxidative, antiviral, anticancer, antidiabetic, immunomodulatory, hepatoprotective and analgesic effects. In the present study, an ultrasonic-assisted extraction method was developed for the effective extraction of mangiferin from mango leaves. Some parameters such as ethanol concentration, liquid-to-solid ratio, extraction temperature, and extraction time were optimized by single-factor experiment and response surface methodology. The optimal extraction conditions were 44% ethanol, the liquid-to-solid ratio was 38:1, and extraction for 19.2 min at 60 °C under ultrasound irradiation of 200 W. Under optimal conditions, the yield of mangiferin was 58.46 ± 1.27 mg/g. The results obtained are helpful for the full utilization of mango leaves, and also indicated that ultrasonic-assisted extraction is a very useful method for the extraction of mangiferin from plant materials.

## 1. Introduction

**Figure 1:**
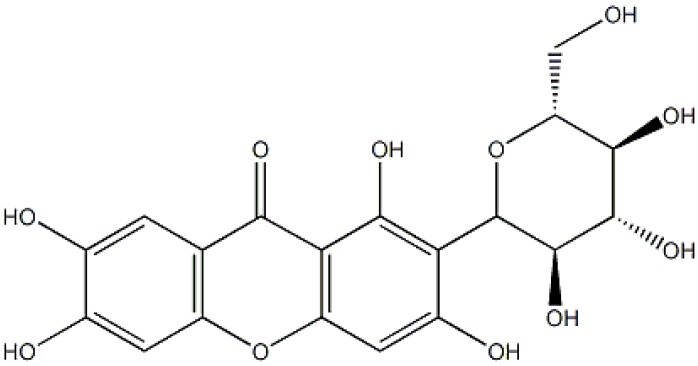
Structure of mangiferin.

Mango (*Mangifera indica* L.), the most important fruit in the *Anacardiaceae* family, is a tropical fruit with high nutritional and medicinal value. Mango leaves is a famous Traditional Chinese Medicine used for prevention and treatment of neuropathic pain and ulcer [1,2,3]. It contains a variety of bioactive compounds, mangiferin being the main active component among them. Mangiferin has attracted a lot of attention because of its various biological activities, such as antioxidative, antiviral, anticancer, antidiabetic, immunomodulatory, hepatoprotective and analgesic effects [4,5,6,7,8,9,10]. The chemical structure of mangiferin is shown in Figure 1.

The active ingredients of mango leaves could be extracted by several ways, such as maceration extraction, solid phase microextraction and hydrodistillation [11,12]. However, these conventional extraction methods are solvent- and time-consuming. Recent studies of novel and green extraction techniques have been developed for extracting active components from plants, such as ultrasonic-assisted extraction (UAE), supercritical and subcritical fluid extraction [13,14,15]. Among these, UAE is an efficient extraction method, which may enhance the extraction efficiency due to the effect of acoustic cavitations produced in the solvent by the passage of an ultrasound wave [16,17]. Ultrasound also exerts a mechanical effect, which allows greater penetration of solvent into the tissue and increases the contact surface area between the solid and liquid phase. Finally, the solute quickly diffuses from the solid phase to solvent [18]. Therefore, UAE has been successfully applied to extract many natural products [19,20,21]. However, the study of response surface methodology for ultrasonic-assisted extraction of mangiferin from mango leaves has not been reported.

Response surface methodology (RSM), originally described by Box and Wilson, is effective for responses influenced by many factors and their interactions [22]. It has been successfully used for optimization of the total flavonoid compound extraction from many medicinal plants [23]. In the present study, mangiferin was extracted by UAE and quantified by high-performance liquid chromatography with ultraviolet detection (HPLC-UV). The effects of several experimental parameters such as ethanol concentration, liquid-to-solid ratio and extraction time on the extraction efficiency of mangiferin from mango leaves were optimized by RSM. The crude extract obtained could be used either in some complex traditional medicines or the further purification of mangiferin. Thus, the results obtained are helpful for the full utilization of mango leaves.

## 2. Results and Discussion

### 2.1. Effect of Ethanol Concentration on the Yield of Mangiferin

**Figure 2:**
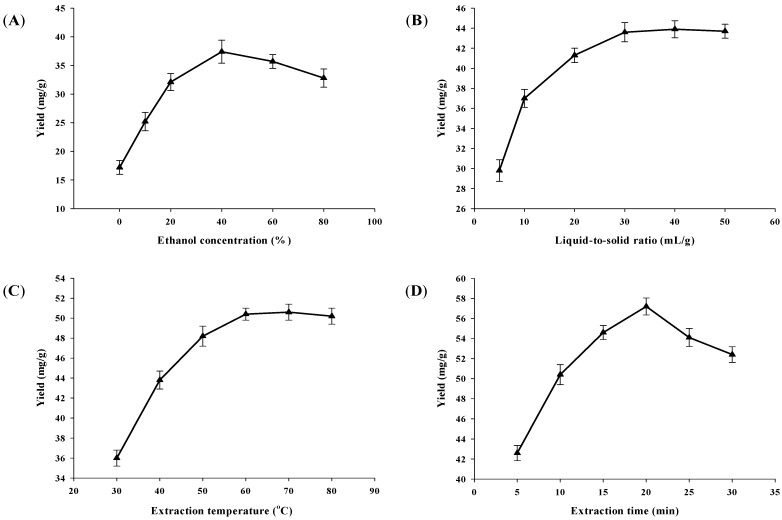
Effects of extraction parameters on the mangiferin yield: (**A**) Effect of ethanol concentration on the yield of mangiferin; (**B**) Effect of liquid-to-solid ratio on the yield of mangiferin; (**C**) Effect of extraction temperature on the yield of mangiferin; and (**D**) Effect of extraction time on the yield of mangiferin.

Generally, methanol, ethanol and water are commonly used extraction solvents. However, methanol is a more toxic solvent than ethanol to human beings and the environment. Water is a nontoxic and inexpensive solvent and has widely been applied for extraction of bioactive compounds in Chinese traditional medicines, but its extraction efficiency is always lower than with a mixture of ethanol and water. Thus, aqueous ethanol was used in this study. The effect of ethanol concentration on the yield of mangiferin was evaluated, and other experimental parameters were set as follows: the liquid-to-solid ratio, 10:1 (mL/g); extraction temperature, 40 °C; and extraction time, 10 min. The results are shown in Figure 2A. When the concentration of ethanol increased from 0 to 40%, the yield of mangiferin increased significantly, reaching a maximum value, which was 37.41 ± 1.90 mg/g, at 40% ethanol, followed by an obvious decrease with further increases of the ethanol concentration from 40% to 80%. The yields of mangiferin extracted by 60% and 80% ethanol were 35.74 ± 1.18 mg/g and 32.82 ± 1.45 mg/g, respectively. The results supported previous findings that a mixture of ethanol and water was a recommendable solvent for the extraction of bioactive components and 40% ethanol was used in the subsequent experiments.

### 2.2. Effect of Liquid-to-Solid Ratio on the Yield of Mangiferin

The effect of liquid-to-solid ratio on the yield of mangiferin was investigated, and other experimental parameters were set as follows: ethanol concentration, 40%; extraction temperature, 40 °C; and extraction time, 10 min. The results are shown in Figure 2B. When the liquid-to-solid ratio increased from 5:1 to 30:1, the yield of mangiferin increased with the increase of the liquid-to-solid ratio. When the liquid-to-solid ratio increased from 30:1 to 50:1, the yield of mangiferin was almost unchanged as the the liquid-to-solid ratio increased. The maximum yield of mangiferin was obtained at a liquid-to-solid ratio of 30:1. Generally, the large liquid-to-solid ratio can dissolve constituents more effectively, leading to an enhancement of the extraction yield [24]. However, this will cause solvent waste. On the contrary, a small liquid-to-solid ratio will result in a lower extraction yield [25]. Therefore, the choice of a proper solvent volume is significant. In summary, the yield of mangiferin increased significantly when the liquid-to-solid ratio increased from 5:1 to 30:1. After 30:1, the mangiferin yield almost unchanged, therefore, a liquid-to-solid ratio of 30:1 was used in the subsequent experiments.

### 2.3. Effect of Extraction Temperature on the Yield of Mangiferin

The effect of extraction temperature on the yield of mangiferin was investigated, and other experimental parameters were set as follows: ethanol concentration, 40%; the liquid-to-solid ratio, 30:1; and extraction time, 10 min. The results are shown in Figure 2C. The yield was improved when the extraction temperature was raised from 30 to 60 °C, and then the yield was almost unchanged from 60 to 80 °C. The highest yield, which was 50.41 ± 0.63 mg/g, could be obtained at 60 °C. Generally, increasing temperature of extraction medium can increase the diffusivity of the solvent into cells and enhances the desorption and solubility of compounds from the cells, which results in the dissolution of components [26]. Although some bioactive compounds from plants could be decomposed at high temperature [17,27], the yield of mangiferin was almost unchanged from 60 to 80 °C. The results indicate that the extraction of mangiferin from plant cells reached an equilibrium of desorption and solubility at 60 °C, and it is a thermal stable component from 60 to 80 °C. Therefore, 60 °C was used in the subsequent experiments.

### 2.4. Effect of Extraction Time on the Yield of Mangiferin

The effect of extraction time on the yield of mangiferin was investigated, and other experimental parameters were set as follows: ethanol concentration, 40%; liquid-to-solid ratio, 30:1; and extraction temperature, 60 °C. The results are shown in Figure 2D. There was an increase in the yield of mangiferin from 5 to 20 min, then it decreased when the irradiating time was longer than 20 min. The maximum yield, which was 57.20 ± 0.85 mg/g, could be obtained at 20 min. The results indicate that ultrasound might accelerate the establishment of an equilibrium for dissolution of the target compounds between the plant cell wall and the extraction solvent in a short time. This is a huge advantage of UAE compared to conventional extraction methods [28]. Mangiferin could be degraded after a long exposure to ultrasonic irradiation, causing the yield to decrease. Therefore, 20 min was chosen as optimal extraction time.

### 2.5. Optimization of the Mangiferin Yield

The yield of mangiferin was further optimized using the RSM approach. A fixed extraction temperature (60 °C) was chosen. The coded and actual levels of the three variables in Table 1 were selected to maximize the yield.

**Table 1:** Coded and actual levels of three variables.

Independent Variables	Coded Levels		
	−1	0	1
Ethanol concentration (*X*_1_, %)	20	40	60
Liquid-to-Solid ratio (*X*_2_, mL/g)	20:1	30:1	40:1
Extraction time (*X*_3_, min)	15	20	25

Table 2 shows the treatments with coded levels and the experimental results of mangiferin yield in mango leaves. The yields ranged from 47.12 to 58.06 mg/g. The maximum yield was recorded under the experimental conditions of *X*_1_ = 40%, *X*_2_ = 30:1 and *X*_3_ = 20 min. By applying multiple regression analysis to the experimental data, the response variable and the test variables are related by the following second-order polynomial Equation (1):

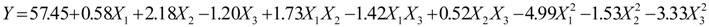
(1)


**Table 2:** Experimental designs using Box-Behnken and results.

Treatment No.	Coded Levels			Mangiferin Yield (mg/g)
	*X*_1_	*X*_2_	*X*_3_	
1	0	1	1	54.03
2	0	−1	−1	52.19
3	−1	−1	0	49.87
4	0	−1	1	48.72
5	1	0	−1	52.35
6	0	0	0	58.06
7	1	0	1	47.12
8	−1	1	0	50.87
9	1	1	0	55.45
10	0	0	0	57.13
11	−1	0	−1	48.31
12	0	0	0	57.27
13	1	−1	0	47.52
14	−1	0	1	48.76
15	0	0	0	56.84
16	0	1	−1	55.41
17	0	0	0	57.93

In the preceding equation, *X*_1_ is the ethanol concentration, *X*_2_ is the liquid-to-solid ratio, and *X*_3_ is the extraction time. Table 3 shows the analysis of variance (ANOVA) for the regression equation. The linear term and quadratic term were very significant (*p* < 0.01). The lack of fit was used to verify the adequacy of the model and was not significant (*p* > 0.05), indicating that the model fit the experiment data adequately.

**Table 3:** Analysis of variance (ANOVA) for the regression equation.

Source	Sum of Squares	Degrees of Freedom	Mean Square	*F* Value	*p* Value
Model	249.40	9	27.91	171.12	<0.0001
*X*_1_	2.68	1	2.68	16.55	0.0048
*X*_2_	38.11	1	38.11	235.32	<0.0001
*X*_3_	11.59	1	11.59	71.58	<0.0001
*X*_1_*X*_2_	12.01	1	12.01	74.14	<0.0001
*X*_1_*X*_3_	8.07	1	8.07	49.81	0.0002
*X*_2_*X*_3_	1.09	1	1.09	6.74	0.0356
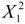	104.65	1	104.65	646.27	<0.0001
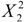	9.90	1	9.90	61.11	0.0001
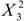	46.56	1	46.56	287.55	<0.0001
Residual	1.13	7	0.16		
Lack of fit	0.024	3	0.0081	0.029	0.9923

The adequate precision measures the signal to noise ratio. A ratio greater than 4 is desirable. In this study, the ratio was found to be 33.55, indicating that this model can be used to navigate the design space. The relationship between the experimental values and predicted values showed that the plotted points cluster around the diagonal line, indicating good fitness of the model because the value of predicted *R*-squared of 0.9915 is in reasonable agreement with the adjusted *R*-squared of 0.9897. A very low value of coefficient of the variance (C.V.%, 0.76) clearly indicated a very high degree of precision and reliability of the experimental values.

**Figure 3:**
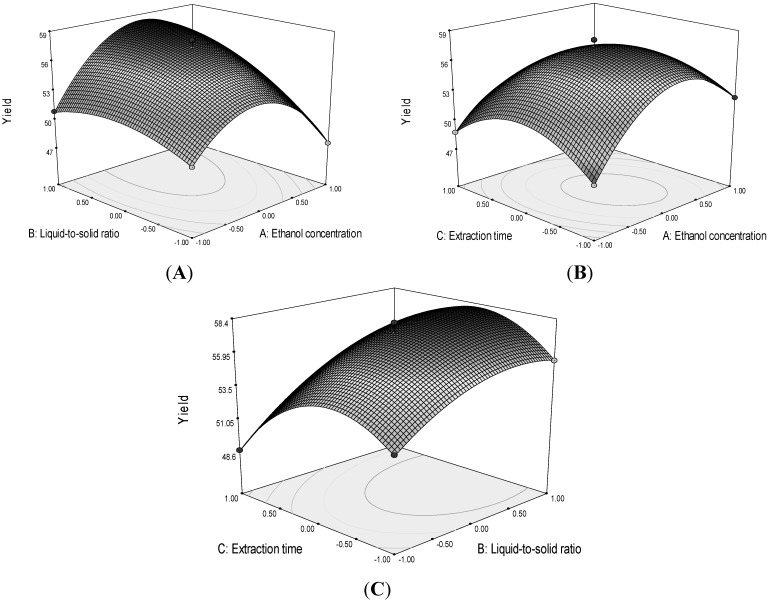
Response surface plots of the mangiferin of mango leaves as affected by ethanol concentration, liquid-to-solid ratio, and extraction time in UAE. (**A**) ethanol concentration (*X*_1_) and liquid-to-solid ratio (*X*_2_); (**B**) ethanol concentration (*X*_1_) and extraction time (*X*_3_); and (**C**) liquid-to-solid ratio (*X*_2_) and extraction time (*X*_3_).

Figure 3 shows the three-dimensional response surface plots. An increase of liquid-to-solid ratio *(X*_2_) results in an increase of mangiferin yield to a maximum at a certain level, while an increase of ethanol concentration (*X*_1_) and extraction time (*X*_3_) result in an initial increase of mangiferin yield that then decreases as the concentration and time continue to increase. The optimal values of the selected variables were obtained by solving the regression equation. After calculation by the Design Expert software, the optimal conditions for extracting mango leaves mangiferin were 44% ethanol, liquid-to-solid ratio was 38:1, and extraction time was 19.2 min, with the corresponding *Y* = 58.49 mg/g. To confirm the result, tests were performed in triplicate under optimized conditions. The yield of mangiferin was 58.46 ± 1.27 mg/g, significantly higher than that of microwave-assisted extraction (36.10 mg/g) [29], which clearly showed that the model fitted the experimental data and optimized the extraction procedure of mangiferin from mango leaves.

## 3. Experimental

### 3.1. Chemicals and Reagents

Mangiferin standard was purchased from Sigma-Aldrich (St. Louis, MO, USA). Ethanol, methanol and phosphoric acid were HPLC grade and bought from Merck (Darmstadt, Germany). Deionized water was prepared using a Milli-Q water purification system and used throughout the experiments.

### 3.2. Plant Material

Mango (*Mangifera indica* L. cv. Tainung No. 1) leaves were collected from the Northern Campus of Sun Yat-sen University, Guangzhou, Guangdong Province, China. The samples were then dried at room temperature, ground into fine powder, and stored at −80 °C.

### 3.3. Ultrasound-Assisted Extraction

The ultrasound-assisted extraction (UAE) was carried out in an ultrasonic device (KJ1004B, Kejin Instrument Company, Guangzhou, China) with an ultrasound power of 200 W and frequency of 40 kHz, equipped with a temperature controller and a digital timer.

The mango leaves powder (1.0 g) was accurately weighed and placed in a capped tube, then mixed with a suitable amount of extraction solvent. After wetting the plant material, the tube with the suspension was immersed into water in the ultrasonic device, and irradiated under the predetermined conditions. After ultrasonic extraction, the sample was centrifuged at 8,000 *g* for 10 min and the supernatant was collected. The precipitation was taken back and extracted again under the same conditions. The extracts of the twice-extraction were mixed and filtered through a 0.45 μm syringe filter (Pall Life Sciences, Ann Arbor, MI, USA) for HPLC analysis.

### 3.4. Experimental Design

Response surface methodology (RSM) was employed to optimize the UAE of mangiferin from mango leaves. A Box-Behnken experiment was employed in this regard. Ethanol concentration (*X*_1_), liquid-to-solid ratio (*X*_2_), and extraction time (*X*_3_) were chosen for independent variables. The range and center point values of the three independent variables presented in Table 1 are selected on the basis of preliminary experiments. The experimental design consists of 12 factorial experiments and five replicates of the central point. Mangiferin yield was selected as the responses for the combination of the independent variables given in Table 2. Experimental runs were randomized, to minimize the effects of unexpected variability on the observed responses. The variables were coded according to the following Equation (2):

*x* = (*X_i_* − *X_0_*) / Δ*X* (2)

where *x* is the coded value, *X_i_* is the corresponding actual value, *X_0_* is the actual value in the center of the domain, and Δ*X* is the increment of *X_i_* corresponding to a variation of 1 unit of *x*. The mathematical model corresponding to the Box-Behnken design is:

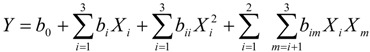
(3)
where *Y* is the predicted response, *b_0_* is a constant, *b_i_*, *b_ii_* and *b_im_* are the model coefficients. They represent the linear, quadratic and interaction effects of the variables. The adequacy of the model was determined by evaluating the lack of fit, coefficient of determination (*R^2^*) and the Fisher test value (*F-value*) obtained from the analysis of variance (ANOVA) that was generated by the Design Expert software.

### 3.5. HPLC Analysis

Mangiferin was analyzed by an Agilent 1200 HPLC system (Agilent, Palo Alto, CA, USA) coupled with an UV detector. An elite^®^ C18 column (250 mm × 4.6 mm, 5 μm) was used. The mobile phase consisted of methanol and 0.1% phosphoric acid in water (30:70, v/v) at a flow rate of 1.0 mL/min [30]. The wavelength of detection was 258 nm, column temperature was 25 °C, injection volume was 20 µL. Mangiferin was quantified based on peak area and comparison with the standard curve.

### 3.6. Statistical Analysis

All experiments were conducted in triplicate, and the results were expressed as the mean ± standard deviation. Analysis of the experimental design data and calculation of predicted responses were carried out using Design Expert software (Version 7.1.6, Stat-Ease, Inc., Minneapolis, MN, USA). Differences were considered significant if *p* < 0.05.

## 4. Conclusions

In the present study, an ultrasonic-assisted extraction method has been developed for the extraction of mangiferin from mango leaves. Ultrasonic waves are a powerful tool which can efficiently improve the extraction yield. RSM was successfully employed to optimize the extraction and several experimental parameters have been evaluated. The results showed that ethanol concentration, liquid-to-solid ratio, and extraction time all had significant effects on the yield of mangiferin extraction. The best combination of response functions was 44% ethanol, a liquid-to-solid ratio of 38:1, and an extraction of 19.2 min at 60 °C under ultrasound irradiation of 200 W. Under the optimal conditions, the yield of mangiferin reached 58.46 ± 1.27 mg/g. The results obtained are helpful for the full utilization of mango leaves, and also confirmed that ultrasonic-assisted extraction is a powerful tool for the extraction of important phytochemicals from plant materials.
